# Age and Sex Differences in the Genetics of Cardiomyopathy

**DOI:** 10.1007/s12265-023-10411-8

**Published:** 2023-07-21

**Authors:** Oyediran Akinrinade, Robert Lesurf, Genomics England Research Consortium, J. C. Ambrose, P. Arumugam, M. Bleda, F. Boardman-Pretty, C. R. Boustred, H. Brittain, M. J. Caulfield, G. C. Chan, T. Fowler, A. Giess, A. Hamblin, S. Henderson, T. J. P. Hubbard, R. Jackson, L. J. Jones, D. Kasperaviciute, M. Kayikci, A. Kousathanas, L. Lahnstein, S. E. A. Leigh, I. U. S. Leong, F. J. Lopez, F. Maleady-Crowe, L. Moutsianas, M. Mueller, N. Murugaesu, A. C. Need, P. O‘Donovan, C. A. Odhams, C. Patch, D. Perez-Gil, M. B. Pereira, J. Pullinger, T. Rahim, A. Rendon, T. Rogers, K. Savage, K. Sawant, R. H. Scott, A. Siddiq, A. Sieghart, S. C. Smith, A. Sosinsky, A. Stuckey, M. Tanguy, E. R. A. Thomas, S. R. Thompson, A. Tucci, E. Walsh, M. J. Welland, E. Williams, K. Witkowska, S. M. Wood, Jane Lougheed, Tapas Mondal, John Smythe, Luis Altamirano-Diaz, Erwin Oechslin, Seema Mital

**Affiliations:** 1 Genetics and Genome Biology Program, The Hospital for Sick Children, 555 University Avenue, Toronto, ON M5G 1X8 Canada; 2 St. George’s University School of Medicine, St. George’s, West Indies Grenada; 3 Division of Cardiology, Children’s Hospital of Eastern Ontario, Ottawa, ON Canada; 4 Division of Cardiology, Department of Pediatrics, McMaster Children’s Hospital, Hamilton, ON Canada; 5 Division of Cardiology, Department of Pediatrics, Kingston General Hospital, Kingston, ON Canada; 6 Division of Cardiology, Department of Pediatrics, London Health Sciences Centre, London, ON Canada; 7 Division of Cardiology, Toronto Adult Congenital Heart Disease Program at Peter Munk Cardiac Centre, Department of Medicine, University Health Network, and University of Toronto, Toronto, ON Canada; 8 Ted Rogers Centre for Heart Research, Toronto, ON Canada; 9 Division of Cardiology, Department of Pediatrics, The Hospital for Sick Children, University of Toronto, Toronto, ON Canada

**Keywords:** Cardiomyopathy, Genetic, Whole genome sequencing, Age, Sex, Genomic constraints

## Abstract

Cardiomyopathy has variable penetrance. We analyzed age and sex-related genetic differences in 1,397 cardiomyopathy patients (Ontario, UK) with whole genome sequencing. Pediatric cases (n = 471) harbored more deleterious protein-coding variants in Tier 1 cardiomyopathy genes compared to adults (n = 926) (34.6% vs 25.9% respectively, p = 0.0015), with variant enrichment in constrained coding regions. Pediatric patients had a higher burden of sarcomere and lower burden of channelopathy gene variants compared to adults. Specifically, pediatric patients had more *MYH7* and *MYL3* variants in hypertrophic cardiomyopathy, and fewer *TTN* truncating variants in dilated cardiomyopathy. *MYH7* variants clustered in the myosin head and neck domains in children. *OBSCN* was a top mutated gene in adults, enriched for protein-truncating variants. In dilated cardiomyopathy, female patients had a higher burden of z-disc gene variants compared to males. Genetic differences may explain age and sex-related variability in cardiomyopathy penetrance. Genotype-guided predictions of age of onset can inform pre-test genetic counseling.

**Graphical Abstract:**

Pediatric cardiomyopathy patients were more likely to be genotype-positive than adults with a higher burden of variants in *MYH7, MYL3, TNNT2, VCL*. Adults had a higher burden of *OBSCN* and *TTN* variants. Females with dilated cardiomyopathy (DCM) had a higher burden of z-disc gene variants compared to males.

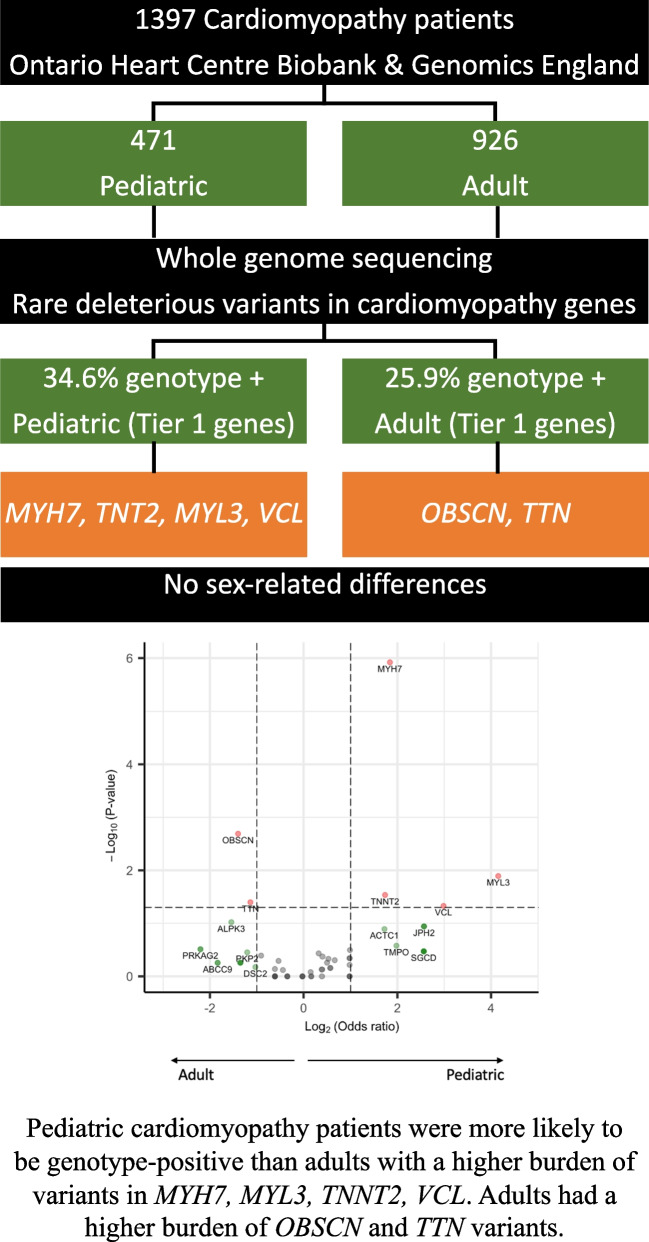

**Supplementary Information:**

The online version contains supplementary material available at 10.1007/s12265-023-10411-8.

## Introduction

Cardiomyopathies are among the most common genetic cardiac disorders and the leading cause of heart failure and sudden cardiac death [1]. Phenotypically, there are five subtypes—dilated (DCM), hypertrophic (HCM), restrictive (RCM), left ventricular non-compaction (LVNC) and arrhythmogenic (ACM) cardiomyopathy. They can be sporadic or familial, and usually have an autosomal dominant inheritance [2–5]. Variants in over 100 genes have been associated with cardiomyopathy, with considerable overlap in genes involved in causing different cardiomyopathy subtypes [4, 6, 7].

While some individuals manifest clinical cardiomyopathy in childhood, others do not manifest disease until adulthood [8–11]. Studies have also reported sex-related differences in the incidence and severity of cardiomyopathy [12–15]. While age-related penetrance, lifestyle factors, and hormonal differences can explain part of the clinical variability by age and sex [16–18], a systematic comparison of childhood versus adult-onset cardiomyopathy, or of cardiomyopathy in females versus males, using comprehensive genomic evaluation is important. Marston et al. reported a higher yield of sarcomere gene variants in pediatric versus adult HCM [9]. However, their study was limited to patients with HCM, was confounded by the variability in the sizes of gene panels used for clinical testing across patients, only focused on sarcomere gene variants, and did not analyze sex-related differences. Whole genome sequencing can overcome some of these limitations by permitting a standardized and comprehensive exploration of all relevant genes across different cohorts and also allows an exploration of not just single nucleotide variants but also of structural variants.

The goal of our study was to use whole genome sequencing to evaluate age and sex-related differences in the genetics of cardiomyopathy by comparing the frequency and distribution of rare, deleterious variants in known cardiomyopathy genes and within constrained regions of these genes between patients with childhood and adult-onset cardiomyopathy, and between affected males and females.

## Results

The study cohort comprised 1,397 unrelated primary cardiomyopathy patients with whole genome sequencing recruited through (i) the multi-institutional Ontario province-wide Heart Centre Biobank Registry (n = 236), and (ii) the 100,000 Genomes Project accessed through the Genomics England Clinical Interpretation Partnership (version 8) (n = 1,161) [19–23]. Patients with secondary causes of cardiomyopathy (syndromic, neuromuscular, metabolic or mitochondrial, congenital heart disease) were excluded. 471 patients were pediatric (< 18 years old at diagnosis) (236 from the Ontario biobank, 235 from Genomics England), and 926 were adults (all from Genomics England); 871 (62%) were male. By subtype, 768 (55%) had HCM, 435 (31%) had DCM, 116 (8%) had ACM, 58 (4%) had LVNC, and 20 (1%) had RCM. The cohort characteristics are described in Table 1. The pediatric cohort had a higher proportion of DCM, LVNC and RCM compared to the adult cohort. Written informed consent was obtained from all biobank participants and/or their parents or legal guardians and the research protocol was approved by the Institutional Research Ethics Boards at all participating sites. The full methods are available as Supplementary Material.

**Table 1:** Characteristics of the study cohort (n = 1,397)

	All (n = 1397)	Pediatric (n = 471)	Adult (n = 926)	*P*-value
Mean age at diagnosis (years)	31.4 ± 21.8	4.8 ± 5.9	44.0 ± 13.7	2.2 × 10^–16^
Male, n (%)	871 (62.3)	284 (60.3)	587 (63.4)	0.28
Subtypes, n (%)				
HCM	768 (55.0)	223 (47.3)	545 (58.9)	5.6 × 10^–5^
DCM	435 (31.1)	175 (37.2)	260 (28.1)	6.7 × 10^–4^
ACM	116 (8.3)	20 (4.2)	96 (10.4)	1.4 × 10^–4^
LVNC	58 (4.2)	33 (7.0)	25 (2.7)	2.4 × 10^–4^
RCM	20 (1.4)	20 (4.2)	-	
Ethnicity, n (%)				
European	851 (60.9)	313 (66.5)	538 (58.1)	2.5 × 10^–3^
South Asian	454 (32.5)	102 (21.7)	352 (38.0)	4.1 × 10^–10^
East Asian	24 (1.7)	16 (3.4)	8 (0.9)	1.5 × 10^–3^
Admixed American	35 (2.5)	15 (3.2)	20 (2.2)	2.8 × 10^–1^
African	33 (2.4)	25 (5.3)	8 (0.9)	7.1 × 10^–7^
n (%) of variant-positive* patients in all genes				
All cardiomyopathy (n = 1397)	588 (42.1)	213 (45.2)	375 (40.1)	0.10
HCM (n = 768)	311 (40.5)	106 (47.5)	205 (37.6)	0.01
DCM (n = 435)	199 (45.7)	74 (42.3)	125 (48.1)	0.28
n (%) of variant-positive* patients in Tier 1 genes				
All cardiomyopathy (n = 1397)	403 (28.8)	163 (34.6)	240 (25.9)	8.8 × 10^–4^
HCM (n = 768)	203 (26.4)	77 (34.5)	126 (23.1)	1.5 × 10^–3^
DCM (n = 435)	141 (32.4)	57 (32.6%)	84 (32.3%)	1

^*^Variant positive = presence of a rare, deleterious variant in a cardiomyopathy gene

HCM, hypertrophic cardiomyopathy; DCM, dilated cardiomyopathy; ACM, arrhythmogenic cardiomyopathy; LVNC, left ventricular non-compaction cardiomyopathy; RCM, restrictive cardiomyopathy

## Variant burden in pediatric versus adult cardiomyopathy

We mapped protein-coding single nucleotide variants, indels and copy number variants to 78 cardiomyopathy candidate genes (30 Tier 1, 48 Tier 2) with autosomal dominant inheritance that were manually curated based on published evidence and representation on commercial gene panels (Supplementary Table S1) [24–28].

### Higher frequency of deleterious variants in pediatric cardiomyopathy

Overall, 588 patients (42.1% of the cohort) harbored one or more rare deleterious variants in 72 genes. A majority i.e., 403 (28.8% of the overall cohort) harbored variants in 30 Tier 1 genes (34.6% in pediatric, 25.9% in adults, p = 0.0015) (Online Abstract Figure). When analyzed by cardiomyopathy subtypes, a higher proportion of pediatric HCM cases harbored a deleterious variant compared to adult HCM (34.% vs 23.1% respectively, p = 1.5 × 10^–3^); this difference was not seen in DCM cases. There was no significant difference in the proportion of patients harboring multiple deleterious variants between pediatric (3.8%) and adult (2.7%) cases (p = 0.33). The list of variants is provided in Supplementary Table S2.

### Higher variant constrained coding region (CCR) scores in pediatric cardiomyopathy

**Fig. 1:**
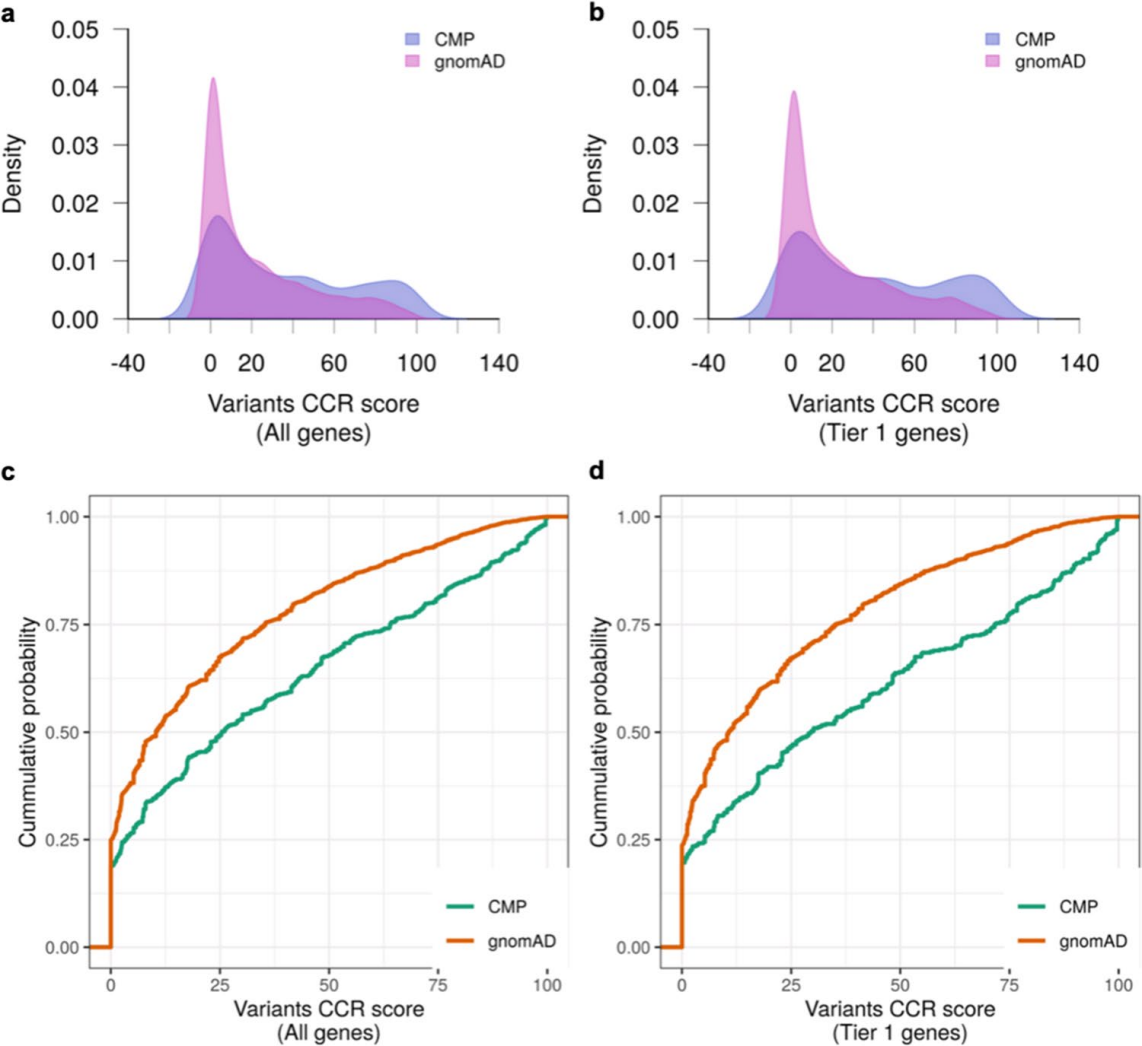
CCR score of deleterious variants in cases and controls. (**a, b**) Density plots of CCR scores (blue = cardiomyopathy; pink = gnomAD), and (**c, d**) Empirical cumulative probability distribution of CCR scores of deleterious variants identified in cardiomyopathy cases (n = 1397) (green) and in gnomAD reference controls (n = 125,748) (orange). Variant CCR scores were higher in cardiomyopathy cases compared to gnomAD reference controls across all cardiomyopathy genes (KS test p < 2.2 × 10^–16^) and in the subset of Tier 1 genes (KS test p < 2.2 × 10^–16^). CCR, constrained coding regions; CMP, cardiomyopathy; gnomAD, Genome Aggregation Database; KS, Kolmogorov–Smirnov test

**Fig. 2:**
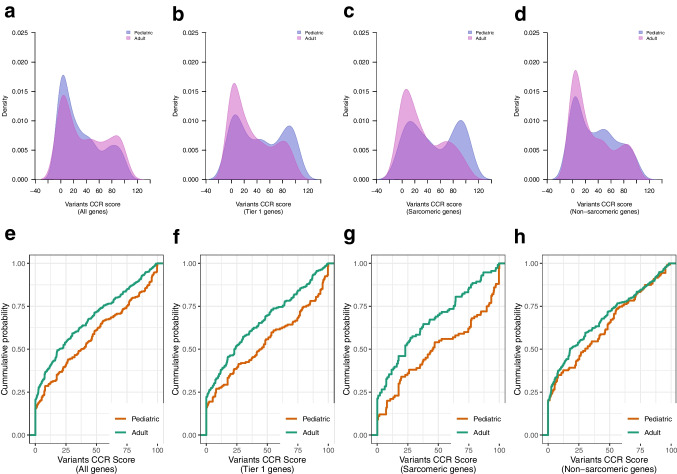
CCR score of deleterious variants stratified by age. (**a-d**) Density plots of CCR scores (blue = pediatric; pink = adult cardiomyopathy), and (**e–h**) Empirical cumulative probability distribution of CCR scores of deleterious variants identified in pediatric (n = 471) (orange) and adult cardiomyopathy cases (n = 921) (green). Variant CCR scores were higher in pediatric versus adult cardiomyopathy cases across all cardiomyopathy genes (KS test p = 0.0025), in the subset of Tier 1 genes (p = 0.0047) and in sarcomere genes (p = 0.0048), but not different in non-sarcomere genes (p = 0.096). CCR, constrained coding regions; CMP, cardiomyopathy; KS, Kolmogorov–Smirnov test

We obtained the CCR score for all deleterious variants identified in cardiomyopathy genes in our study cohort and in gnomAD. Variant CCR scores were compared between cases and reference control genomes available through the Genome Aggregation Database (gnomAD v2.1) (n = 125,748) using Kolmogorov–Smirnov (KS) two-tailed test. As expected, deleterious variants identified in cardiomyopathy patients were in more constrained coding regions compared to those in the gnomAD reference population i.e., had higher CCR scores (KS test p < 2.2 × 10^–16^) (Fig. 1a, c). This difference was also seen in the subset of Tier 1 genes (KS test p < 2.2 × 10^–16^) (Fig. 1b, d). For genes harboring deleterious variants, we generated variant CCR score density maps (Figs. 2a-d) and compared CCR score probability distribution using KS two-tailed test (Figs. 2e-h). Variant CCR scores across all cardiomyopathy genes were higher in pediatric compared to adult cases (KS test p = 0.0025) and also when limited to variants in Tier 1 genes (KS test p = 0.0047). CCR scores of variants in sarcomere genes were also higher in pediatric versus adult cases (KS test p = 0.0048). CCR scores of variants in non-sarcomere genes did not differ between pediatric and adult cases (KS test p = 0.096). Overall, these findings suggest that those with early onset disease were more likely to harbor variants within highly constrained coding regions of sarcomere genes.

## Affected genes in pediatric and adult cardiomyopathy

### Single nucleotide variants and indels

**Fig. 3:**
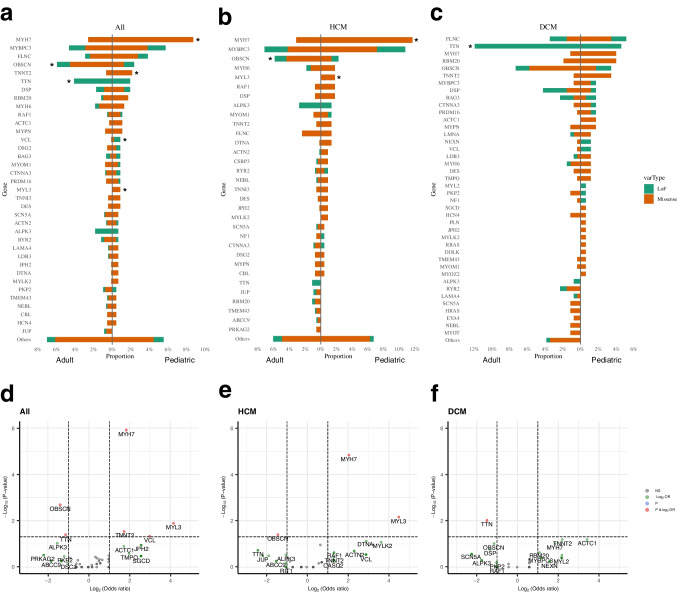
Genes harboring deleterious variants stratified by age. Bar plots showing genes harboring deleterious variants in patients with (**a**) any cardiomyopathy (n = 1397), (**b**) only hypertrophic cardiomyopathy (n = 768), and (**c**) only dilated cardiomyopathy (n = 435). *p < 0.05 between pediatric and adult patients by Fisher test. (**d-f**) Volcano plots showing log odds ratio of the frequency of variants between pediatric and adult patients with (**d**) any cardiomyopathy, (**e**) hypertrophic cardiomyopathy only, and (**f**) dilated cardiomyopathy only. *MYH7, TNNT2, MYL3* and *VCL* were more frequently mutated in pediatric patients, and *TTN* and *OBSCN* were more frequently mutated in adult patients. (orange bar = missense variants, green bar = loss of function variants). NS, no significant difference (grey); log2 OR, genes meeting log2 (odds ratio) > 1 between pediatric and adult (green) (vertical lines); p, genes meeting p < 0.05 between pediatric and adult (blue) (horizontal line); p & log2 OR—genes meeting p < 0.05 with at least 1.5 odds ratio (red)

**Fig. 4:**
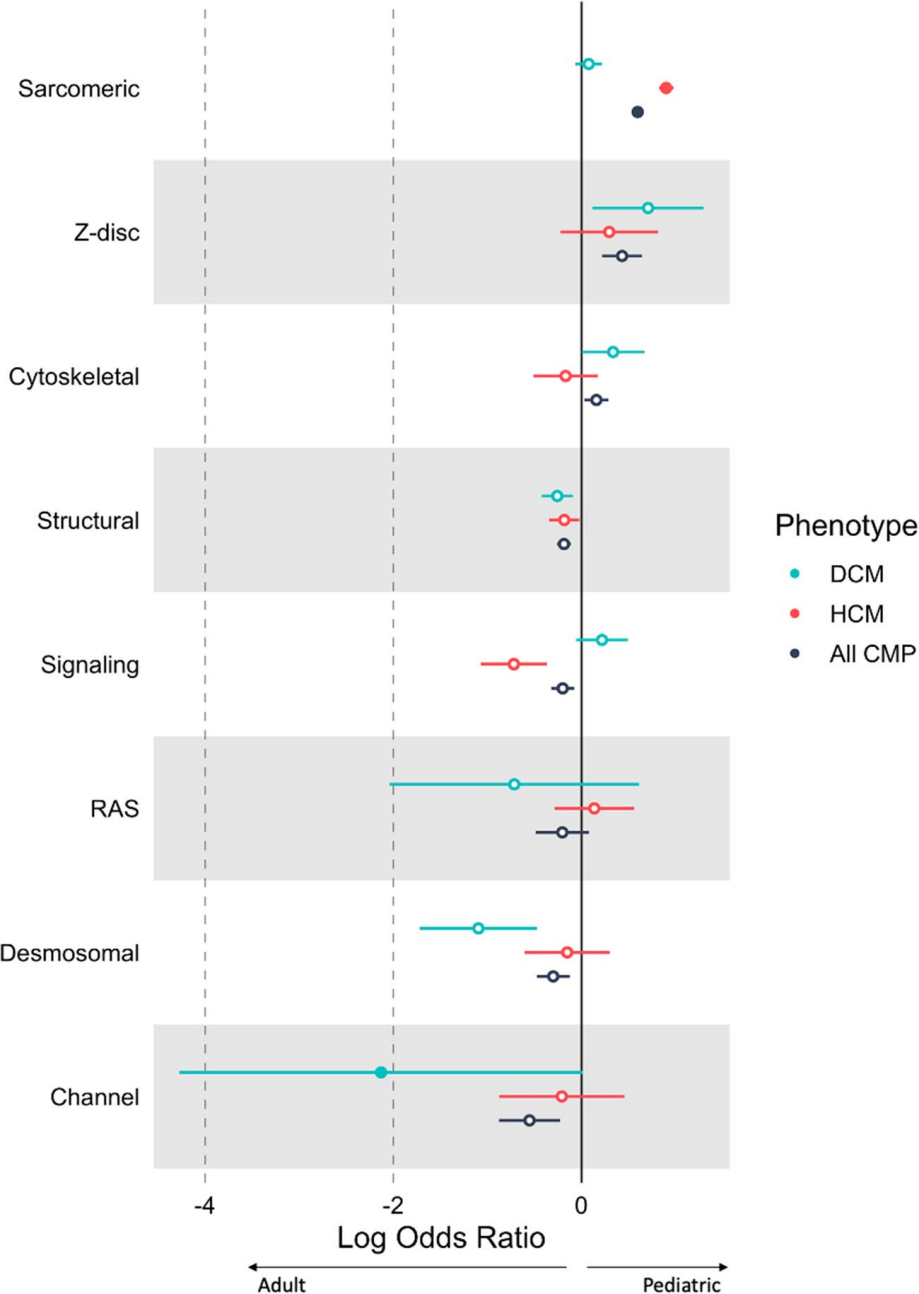
Gene categories harboring rare deleterious variants stratified by age. The forest plot shows the log odds ratio comparing proportion of variant carriers between 471 pediatric and 926 adult patients for different gene categories across cardiomyopathy subtypes. Sarcomere genes were more frequently mutated in pediatric HCM cases while channelopathy genes were more frequently mutated in adult DCM patients. Filled circles indicate significant differences between pediatric and adult patients (p < 0.05). CMP, cardiomyopathy (black); DCM, dilated cardiomyopathy (blue), HCM, hypertrophic cardiomyopathy (red)

For each gene, we compared the proportion of patients harboring at least one deleterious variant in the gene. Figure 3a-c shows the proportion of pediatric and adult patients harboring deleterious variants by gene and by variant types i.e., missense and loss-of-function (LoF). Since there was a difference in cardiomyopathy subtypes between pediatric and adult cases, we analyzed genetic differences by cardiomyopathy subtype. Figures 3d-f show volcano plots by cardiomyopathy subtype for differentially mutated genes between pediatric and adult cases. Pediatric cases had a higher variant burden in *MYH7* (OR 3.58, p = 1.2 × 10^–6^) and *MYL3* (OR 17.8, p = 0.013) but a lower variant burden in *OBSCN* (OR 0.38, p = 0.002). These differences were primarily seen in HCM cases. *TTN* truncating variants were more frequent in adult DCM cases compared to childhood DCM cases (Fig. 3d, Supplementary Table S3). Although *OBSCN* is not currently considered a Tier 1 gene for cardiomyopathy, we found a significantly higher burden of high confidence *OBSCN* LoF variants in our overall cohort compared to gnomAD reference controls (OR = 1.72, CI 1.01–2.75, p = 0.028) (Supplementary Table S4). This enrichment was seen primarily in adult cardiomyopathy patients compared to the gnomAD reference population (p = 0.033). This enrichment of LoF *OBSCN* variants was not seen in pediatric cardiomyopathy patients (p = 0.41 vs gnomAD) (Supplementary Table S4). Another gene that emerged with a significant variant burden in the overall cohort was *VCL*. While overall accounting for fewer than 1% of cases, LoF variants in *VCL* were only seen in children, not in adults (OR = 13.8, p = 0.038). Finally, when genes were collapsed into functional gene categories, pediatric HCM patients had a higher burden of variants in sarcomere genes compared to adult HCM, while pediatric DCM patients had a lower burden of variants in channelopathy genes compared to adults (Fig. 4, Supplementary Table S5).

We performed de novo variant calling in cardiomyopathy genes in a subset of 125 probands (95 pediatric and 30 adult) with complete trio WGS data. Deleterious de novo variants in known genes (*MYH7, TNNI3, TPM1, TNNT2, TTN, DES, RYR2 and NF1*) were identified in 11 (12%) pediatric probands but no de novo variants were identified in adult-onset cardiomyopathy (OR 8.3; p = 0.042) although the analysis was limited by the relatively small number of complete trio data.

### Structural variants

Whole genome sequencing provides an opportunity to explore additional deleterious variant types that standard panel and exome sequencing have a limited capacity to detect. We investigated whether structural variants, including copy number losses or gains, inversions, or insertions, affected genes that were highly mutated between pediatric and adult cardiomyopathy patients i.e., *MYH7*, *MYL3*, *OBSCN*, *TTNT2*, *TTN*, and *VCL*. This identified six additional predicted deleterious copy number deletions in *OBSCN* and *TTN,* none of which have been previously reported in the reference population. Of these, three were also deemed pathogenic by the American College of Medical Genetics and Genomics criteria (Supplementary Table S6).

## Variant location across protein domains by age

**Fig. 5:**
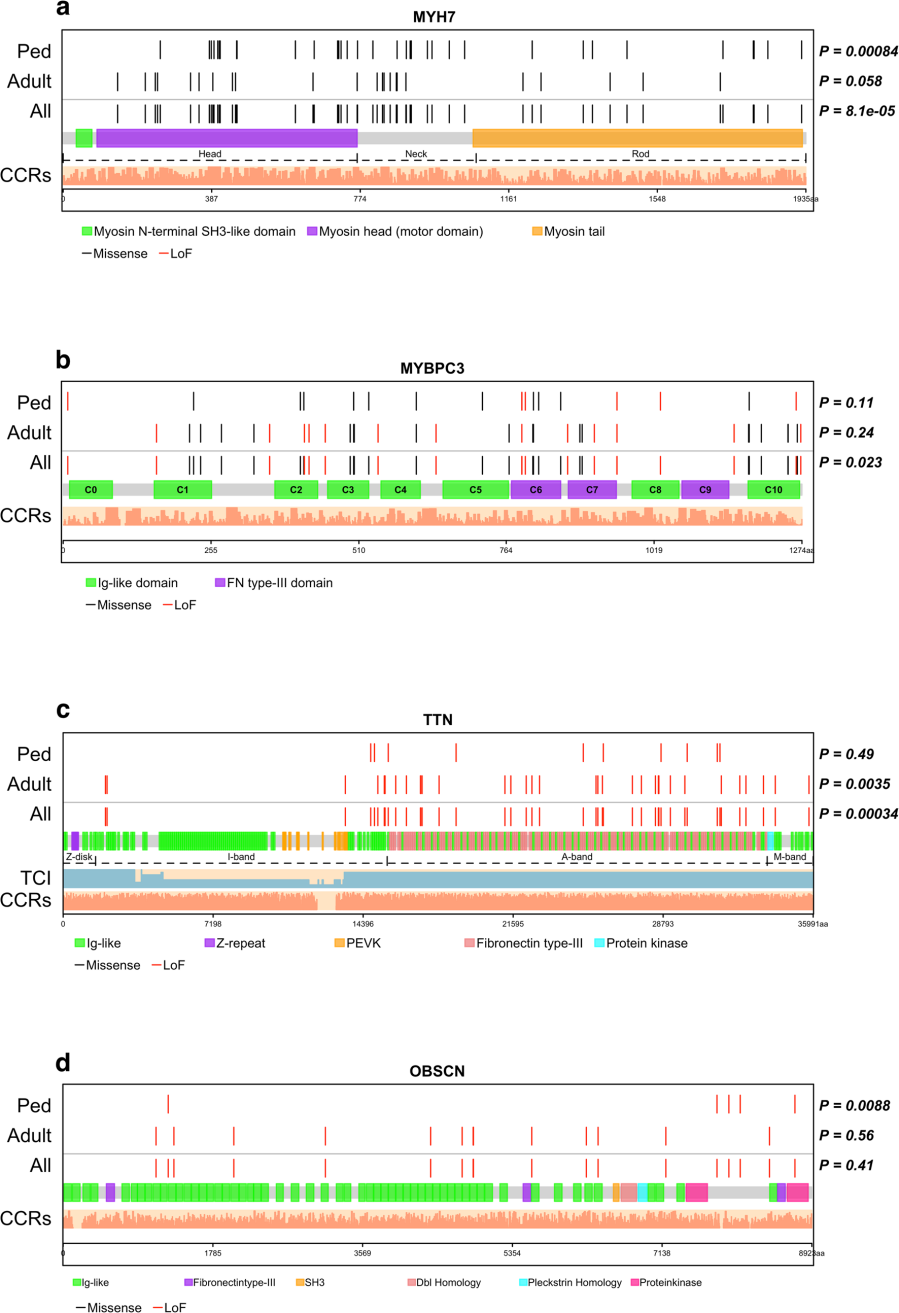
Location of deleterious variants within protein domains. Each protein is linearly depicted with uniprot domain information. Non-random distribution of variants within each protein was assessed using Kolmogorov–Smirnov goodness-of-fit test. Light salmon bars show the constrained coding region scores across the entire protein. (**a**) MYH7 showing head, neck and rod/tail regions. Unlike variants in adult patients, deleterious variants in pediatric patients were non-uniformly distributed with clustering within head and neck domains and fewer variants in the tail domain (p = 0.00084 vs gnomAD distribution). (**b**) MYBPC3 showing the immunoglobulin and fibronectin type 3 domains. Deleterious variants clustered in C5, C7, and C10 domains (p = 0.023), and this distribution did not differ between pediatric (n = 471) and adult (n = 926) patients. (**c**) TTN showing the four regions and all domains. The light blue bars under titin show the transcript count index. *TTN* truncating variants clustered in the A-band domain with non-uniform distribution in adult patients (p = 0.0035). (**d**) OBSCN domains. Deleterious variants clustered around the protein kinase domains of OBSCN in pediatric patients (p = 0.0088). CCR, Constrained coding region; TCI, transcript count index

We used goodness of fit test to determine if deleterious SNVs and indels were uniformly distributed within protein domains of genes or clustered in specific protein domains within genes. This was analyzed only for the top mutated genes in the overall cohort. Figure 5 shows the spatial distribution of deleterious variants in *MYH7, MYBPC3, TTN,* and *OBSCN* against the transcript count index [29]. Deleterious variants were non-uniformly distributed in *MYH7* in pediatric patients with variants clustering within the myosin head and neck domains (KS test p = 8.4 × 10^–4^). In adults, variants were also predominantly in the myosin head and neck domains but were also seen in the tail domain similar to the reference population (KS test, p = 0.058) (Fig. 5a). For *MYBPC3*, variants clustered within the C5, C7, and C10 domains with no difference between children and adults (Fig. 5b). *TTN* truncating variants were non-evenly distributed with clustering in the A-band (p = 3.4 × 10^–4^) in adult DCM patients (p = 3.5 × 10^–3^) [29–31], but not in pediatric cases (p = 0.49) (Fig. 5c). *OBSCN* deleterious variants were non-evenly distributed and clustered around the protein kinase domain of the gene in pediatric patients only (p = 0.0088) (Fig. 5d). When analyzed by cardiomyopathy subtypes, *MYH7* variants in HCM patients clustered within myosin head and neck regions in both pediatric and adult HCM patients but were rare in the head domain in DCM patients (Supplementary Figure S1). Similarly, *TTN* truncating variants clustered within the A-band in both pediatric and adult DCM patients, with non-random distribution in adult DCM (Supplementary Figure S2c).

## Variant burden by sex

**Fig. 6:**
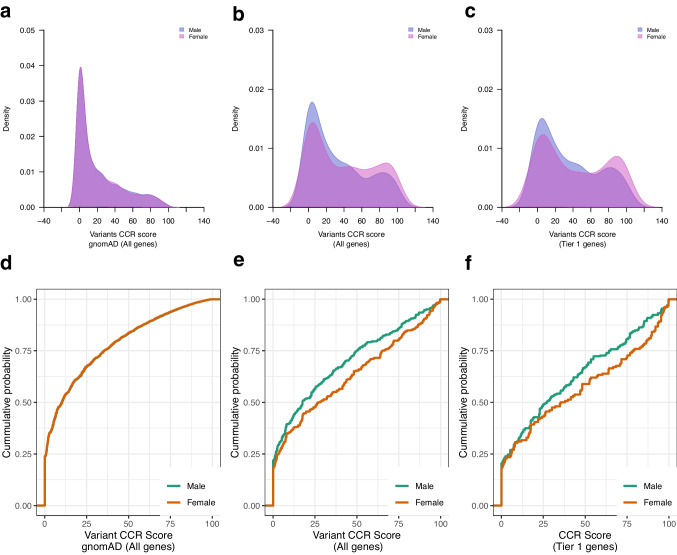
CCR score of deleterious variants in male and female cases and controls. (**a-c**) Density plots of deleterious variant CCR scores (blue = male, pink = female), and (**d-f**) Empirical cumulative probability distribution of deleterious variant CCR scores in 1397 cardiomyopathy cases (871 males, 526 females) and in 125,748 gnomAD reference population stratified by sex (green = male; orange = female). There was no difference in variant CCR scores between males and females (**a, d**) for all genes in gnomAD reference population (p = 0.92), (**b, e**) for all genes in cardiomyopathy cases (KS test p = 0.068), and (**c, f**) for Tier 1 genes in cardiomyopathy cases (p = 0.16). CCR, constrained coding regions; CMP, cardiomyopathy; gnomAD, Genome Aggregation Database

**Fig. 7:**
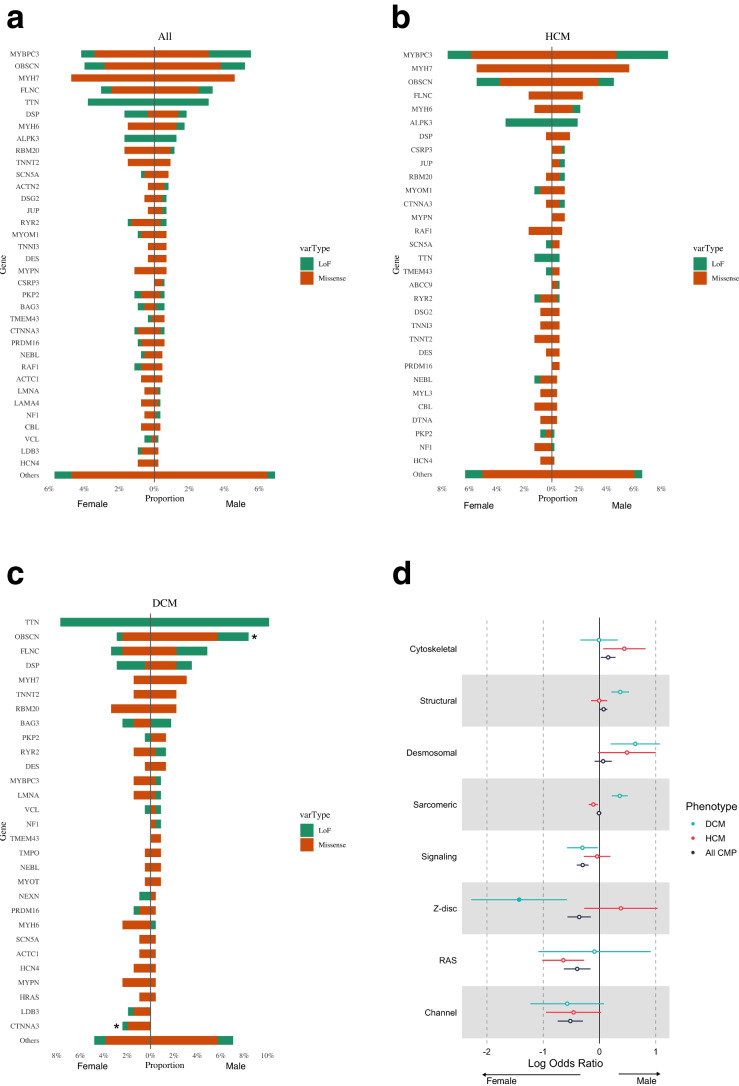
Genes and gene categories harboring deleterious variants stratified by sex. (**a**) Bar plots showing no difference in burden of rare deleterious variants in cardiomyopathy genes between male and female patients (871 males, 526 females) across all cardiomyopathy types, (**b**) in HCM patients, and (**c**) in DCM patients (orange = missense variants, green = loss of function variants). (**d**) The forest plot shows the log odds ratio comparing proportion of variant carriers between male and female patients across different gene categories in DCM, HCM and overall cohort. Z-disc genes were enriched for variants in female compared to male DCM patients. Filled circles indicate significant differences between male and female patients (p < 0.05). CMP, cardiomyopathy (black); DCM, dilated cardiomyopathy (blue), HCM, hypertrophic cardiomyopathy (red); LoF, loss of function

Of 526 females, 228 (43%) were genotype positive, and of 871 males, 355 (41%) were genotype-positive (p = 0.342). There were no sex-related differences in variant CCR scores in gnomAD reference genomes (p = 0.51) (Fig. 6a, d). However, in the cardiomyopathy cohort, the deleterious variant CCR scores were higher in females compared to males. This was true of variants in all cardiomyopathy genes (p = 0.0025) (Fig. 6b, e) as well as for variants in Tier 1 genes (p = 0.019) (Fig. 6c, f). There was no difference in variant burden between sexes across all cardiomyopathies, or in HCM and DCM subtypes (Fig. 7a; Supplementary Table S7). At the gene category level however, variants in z-disc genes were enriched in female DCM patients compared to male DCM patients (p = 0.027) (Fig. 7b; Supplementary Table S8).

## Variant location across protein domains by sex

**Fig. 8:**
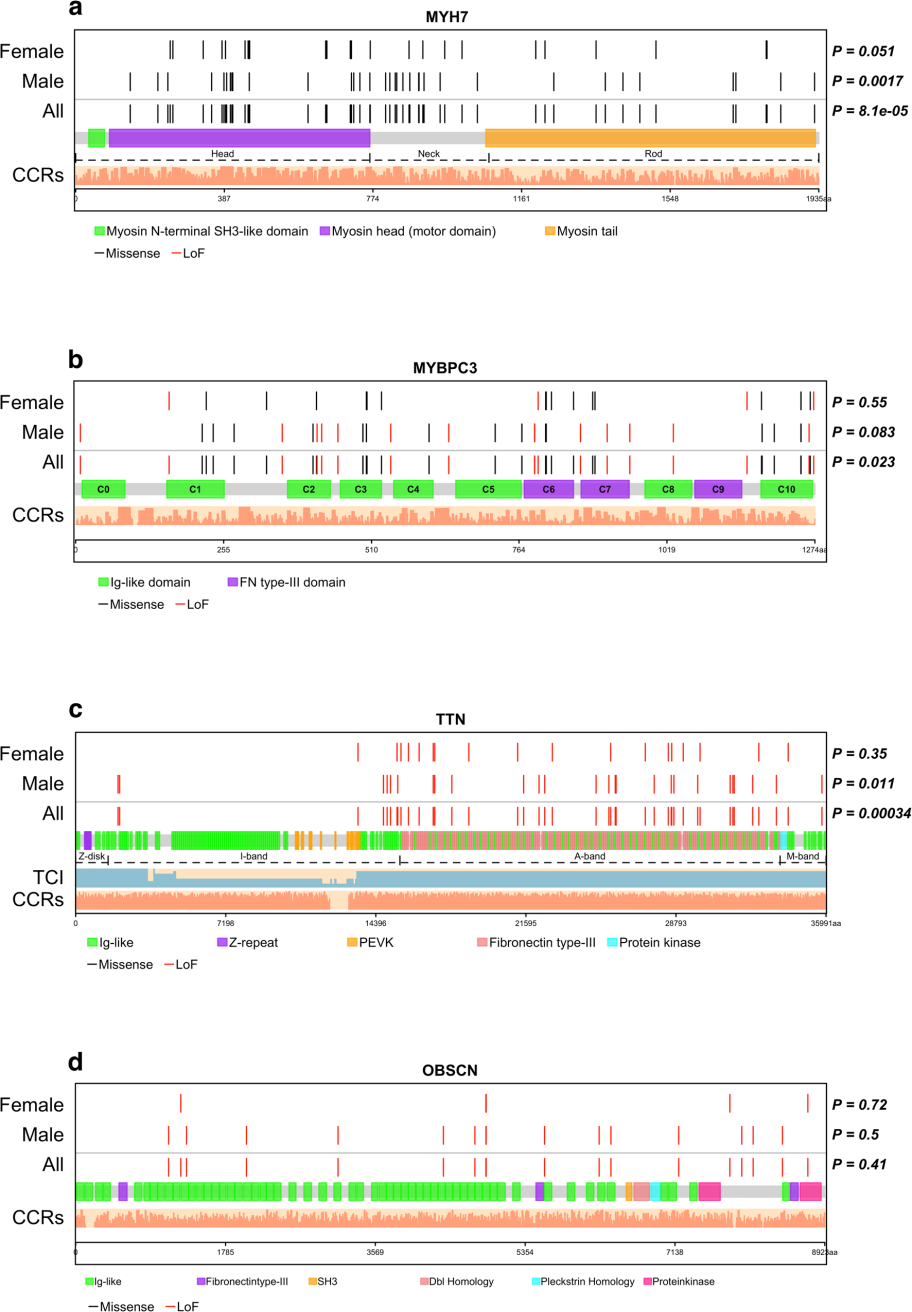
Location of deleterious variants within protein domains stratified by sex. Each protein is linearly depicted with uniprot domain information. Non-random distribution of variants within each protein was assessed using Kolmogorov–Smirnov goodness-of-fit test. Light salmon bars show the constrained coding region scores across the entire protein. (**a**) *MYH7* showing head, neck and rod/tail regions. Deleterious variants were non-uniformly distributed with clustering within head and neck domains and fewer variants in the tail domain. (**b**) *MYBPC3* showing the immunoglobulin and fibronectin type 3 domains. Deleterious variant distribution did not differ between male and female patients. (**c**) *TTN* showing the four regions and all domains. The light blue bars under titin show the transcript count index. *TTN* truncating variants clustered in the A-band with non-uniform distribution in male patients (p = 0.011). (**d**) *OBSCN* LoF variants were uniformly distributed across protein domains in male and female patients. CCR, Constrained coding region; TCI, transcript count index; LoF, loss of function

Figure 8 shows the spatial distribution of deleterious variants in *MYH7, MYBPC3, TTN,* and *OBSCN. MYH7* deleterious variants clustered within myosin head and neck domain in male and female patients but this was significant only in males (KS test p = 0.0017 in males, p = 0.051 in females) likely due to lower numbers of female patients (Fig. 8a). *MYBPC3* deleterious variants were evenly distributed within the protein domains in both male and female patients (Fig. 8b). *TTN* truncating variants were located in the A-band of *TTN* with uneven distribution in male patients (KS test p = 0.01) (Fig. 8c). By cardiomyopathy subtype, *MYH7* deleterious variants clustered within myosin head and neck regions (Supplementary Fig. 3a) and *TTN* truncating variants clustered within the A-band zone in male and female patients (Supplementary Fig. 4).

## Discussion

Our study provides new insights into the genetic architecture of cardiomyopathy. We showed major genetic differences by age of onset of disease and modest differences by sex. The use of whole genome sequencing in this context provided a standardized comparison between groups without being confounded by variable sizes of gene panels used in clinical testing. This helped us identify differences in variant burden by cardiomyopathy type in not only sarcomere but also non-sarcomere genes e.g., *OBSCN* was enriched for variants in adults, while *VCL* was more frequently mutated in children. It also helped us identify deleterious structural variants that would not readily be detected by panel testing or exome sequencing. These findings advance our understanding of the genetics of cardiomyopathy and have implications for using genetic information to guide predictions for age-related disease penetrance.

We included genes associated with cardiomyopathy that are also captured on one or more commercial gene panels. It is important when doing burden analyses to be inclusive since genes with lower penetrance may play a role in the penetrance of late-onset cardiomyopathy. Overall, we found a higher yield of deleterious variants in pediatric HCM patients (34.5%) compared to adult HCM patients (23.1%) especially for variants in Tier 1 genes, consistent with a recent report by Marston et al. (8). We also found a higher burden of de novo variants in cardiomyopathy genes in childhood compared to adult-onset cardiomyopathy. This information could be useful in pre-test counseling regarding diagnostic yield of genetic testing in patients by age of disease onset in the proband. Variants in pediatric patients clustered within more highly constrained coding regions of cardiomyopathy genes compared with those identified in adult patients. This was particularly true of variants in Tier 1 cardiomyopathy genes, including sarcomere genes. This is not entirely unexpected since variants in highly conserved regions are more likely to be intolerant of variation and therefore may manifest earlier penetrance compared to variants in less conserved regions that may show delayed penetrance since they may require an interaction with environmental factors and co-morbidities that usually accumulate with age [32]. The location of variants can also be an important predictor of penetrance as previously reported [33]. Deleterious variants in *MYH7* in childhood HCM cases clustered in the myosin head and neck regions with very few variants seen in the tail domain unlike adult cases. The head and neck regions of MYH7 contain important functional domains i.e., ATPase site, actin binding site, the converter, and essential light chain, that play critical roles in myocardial contraction and energetics, and therefore variants in these regions are likely to cause more severe functional disturbances in encoded protein compared to variants in the tail domain [34–36].

At a gene level, *MYH7* was the most affected gene in pediatric cardiomyopathy affecting 8.7% of cases (compared to 2.6% in adult HCM), while *OBSCN* was the most affected gene in adult HCM affecting 5.9% of adult cases (compared to 2.3% of pediatric HCM cases). A lower frequency of *MYH7* variants in adults compared to the pediatric cohort or compared to findings in the SHaRE cohort may be related in part to differences in race and ethnicity (58% of patients in our study were of European ancestry compared to ~ 90% in SHaRe) [9], or unaccounted for bias towards recruiting genotype-elusive patients in the UK cohort.

Our study further confirmed that truncating variants in the A-band of titin remain the most common genetic cause of adult DCM accounting for 12% of adult cases as opposed to only 4.6% of pediatric DCM cases [29–31, 37]. Recent studies suggest that *TTN* variants may also act as genetic modifiers that predispose to heart failure in other conditions like myocarditis [38, 39]. Interestingly, *OBSCN* emerged as the second most mutated gene in adult DCM accounting for 7.3% of adult cases consistent with a recent report [40], but only 3.4% of pediatric DCM. Similar to TTN which is a giant protein, OBSCN is a giant protein in the sarcomere that interacts with the z-disc region of titin. Protein truncating variants in *OBSCN* were enriched in our cases compared to the gnomAD reference population, primarily in adults, reinforcing its role as a cardiomyopathy associated gene. This is consistent with findings from Wu et al. who showed a significant enrichment of *OBSCN* truncating variants in adult HCM compared to controls [40]. Whole genome sequencing also identified additional deleterious structural variants in *OBSCN* (and *TTN)* that have not been previously reported. At this time, *OBSCN* is considered a gene with limited evidence for association with cardiomyopathy [24, 25] probably due to variable or late penetrance. While most patients in our cohort harboring *OBSCN* variants were genotype-elusive for other Tier 1 genes, it is possible that *OBSCN* variants may act in some patients as a contributing or sensitizing variant as reported previously where *OBSCN* variants often occurred in conjunction with a second disease-associated variant [41]. This may account for the delayed penetrance observed in patients with only *OBSCN* variants. *OBSCN* missense and frameshift variants have been shown to co-segregate with HCM, DCM, and LVNC [40–43]. *OBSCN* deleterious variants in pediatric patients clustered around the protein kinase domains that interact with *CDH2* known to cause ACM [44]. This provides a target for future research towards understanding the mechanism of effect of *OBSCN* variants. Finally, LoF variants in *VCL*, an important cytoskeletal protein with reported association with DCM [45, 46] were only seen in children, not in adults. This is consistent with a previous study that reported *VCL* LoF variants in pediatric DCM [47]. The patients harboring *VCL* variants in our cohort did not harbor any other pathogenic or likely pathogenic variants. Our findings add to the growing evidence for *VCL* LoF as a cause of childhood-onset DCM.

Sex-related phenotype differences in disease frequency and severity have been reported in cardiomyopathy with usually a higher prevalence, earlier onset and greater disease severity in male patients [12–15]. Other studies have reported conflicting findings [16–18]. In a study of HCM patients, female patients were more likely to harbor pathogenic or likely pathogenic variants in sarcomere genes compared to males [48]. In the study by van Velzen et al., there was no difference in genetic yield by sex but there was a lower genetic yield in women over 70 years of age at which age environmental factors may play a larger role than genetic factors [18]. The mean age of female patients in our cohort was 36 years. The higher yield of genetic testing in female patients in our cohort may reflect a cohort enriched for primary genetic cardiomyopathy. Variant CCR scores were also higher in female patients compared to male patients. Additionally, our study showed that z-disc genes were more frequently mutated in female DCM patients. Sex-related differences in genetics may explain some of the differences in clinical presentation and outcomes in cardiomyopathy. However, cardiac sex disparities may also be related to environmental differences or mechanisms regulated by sex hormones, and sex chromosomes [49].

Our findings of genetic differences between childhood and adult-onset cardiomyopathy have potential clinical implications. Children with cardiomyopathy had a higher diagnostic yield for variants in cardiomyopathy genes on sequencing compared to adults. This information can be used to inform pre-test counseling by providing greater precision in communicating the pre-test probability of a positive genetic test result based on age of the affected individual. It can help patients and families in decision making regarding genetic testing. Genetic results in cardiomyopathy patients are widely used for screening asymptomatic family members to identify at-risk individuals. However, it has been difficult to anticipate when clinical disease may manifest in an at-risk family member. While other genomic and environmental factors likely influence disease penetrance, our findings suggest that the genetic cause itself can be an important predictor of age of disease onset. A better understanding of how genetics guides disease penetrance may help in predictive counseling of family members, and in the approach to echocardiographic screening of at-risk family members.

The study has a few limitations. We limited our analysis to coding variants in known cardiomyopathy genes and did not include regulatory non-coding variants, in order to be consistent with what is offered in real world clinical gene panels. We adjusted for differences in race/ethnicity between cohorts but could not eliminate unaccounted for recruitment bias that may have contributed to some of the observed genetic differences. For example, although prior clinical genetic testing was not an exclusion criterion for patient eligibility, the Genomics England cohort may have been biased towards recruiting patients who were negative on previous panel testing. However, since the characteristics of patients not recruited is not available, it is not possible to ascertain if or to what extent there was a recruitment bias. Detailed clinical outcomes were not available in the adult cohort, therefore analysis of association of genotype with outcomes could not be performed. The role of environmental factors, co-morbidities and hormonal influences on age of onset or sex differences could also not be ascertained. Patients with clinically or genetically confirmed syndromes were excluded. Nonetheless, we found RASopathy associated variants in some pediatric and adult HCM patients on whole genome sequencing. We were able to do reverse phenotyping in accessible pediatric patients and found features suggestive of RASopathy in one patient. We did not exclude this patient post-hoc since the diagnosis was made later based on our genetic findings. We were not able to do reverse phenotyping in the UK patients to which we did not have clinical access.

Overall, our study identified a higher variant burden in pediatric cases, especially pediatric HCM compared to adults. The higher genomic constraints on variants found in pediatric patients suggest greater deleterious effects of these variants as a potential mechanism for earlier disease penetrance. Overall, our findings suggest that affected gene, variant location within the gene, and variant constraint scores may be useful in predicting disease penetrance by age and sex. This knowledge may inform pre-test counseling regarding diagnostic yield of genetic testing by patient age and help in predictive counseling of affected families by identifying individuals likely to develop early onset disease.

### Supplementary Information

Below is the link to the electronic supplementary material.

Supplementary file1 (XLSX 119 KB)

Supplementary file2 (DOCX 1038 KB)

Supplementary file3 (DOCX 118 KB)

## Abbreviations

ACM: Arrhythmogenic cardiomyopathy
CCR: Constrained coding region
DCM: Dilated cardiomyopathy
gnomAD: Genome Aggregation Database
HCM: Hypertrophic cardiomyopathy
KS: Kolmogorov-Smirnov
LoF: Loss-of-function
LVNC: Left ventricular non-compaction
OR: Odds Ratio
RCM: Restrictive cardiomyopathy

## Declarations

### Animal Studies

No animal studies were carried out by the authors for this article.

### Data Statement

Sequencing data from the Ontario Registry are deposited in the European Genome-Phenome Archive (EGA) under accession EGAS00001004929, and are available for download upon approval by the Data Access Committee. The 100,000 Genomes Project data are available to researchers and trainees using the Genomics England Research Environment upon institutional approval through their Participation Agreement process. Additional data generated or analyzed during this study are included in the supplementary information files, and additional raw data used for figures and results are available from the corresponding author on reasonable request.
