# Supplementary material for: Age and Sex Differences in the Genetics of Cardiomyopathy
Source: J Cardiovasc Transl Res. 2023 Jul 21;16(6):1287–302. doi: 10.1007/s12265-023-10411-8 (PMC10721711; doi:10.1007/s12265-023-10411-8)
Supplement: Supplementary file 2 — Supplementary file2 (DOCX 1038 KB) [file 12265_2023_10411_MOESM2_ESM.docx]

**SUPPLEMENTARY FIGURES**

**Figure S1. Location of deleterious variants within protein domains** **in HCM stratified by age**. Each protein is linearly depicted with uniprot domain information. Non-random distribution of variants within each protein was assessed using Kolmogorov–Smirnov goodness-of-fit test. Light salmon bars show the constrained coding region scores across the entire protein. (**a**) *MYH7* showing head, neck and rod/tail regions. Deleterious variants were non-uniformly distributed with significant clustering within head and neck domains in pediatric patients. (**b**) *MYBPC3* showing the immunoglobulin and fibronectin type 3 domains. Deleterious variant distribution did not differ between pediatric and adult patients. (**c**) *TTN* showing the four regions and all domains. The light blue bars under titin show the transcript count index. (**d**) *OBSCN* LoF variants were uniformly distributed across protein domains.

HCM, hypertrophic cardiomyopathy; CCR, Constrained coding region; TCI, transcript count index; LoF, loss of function


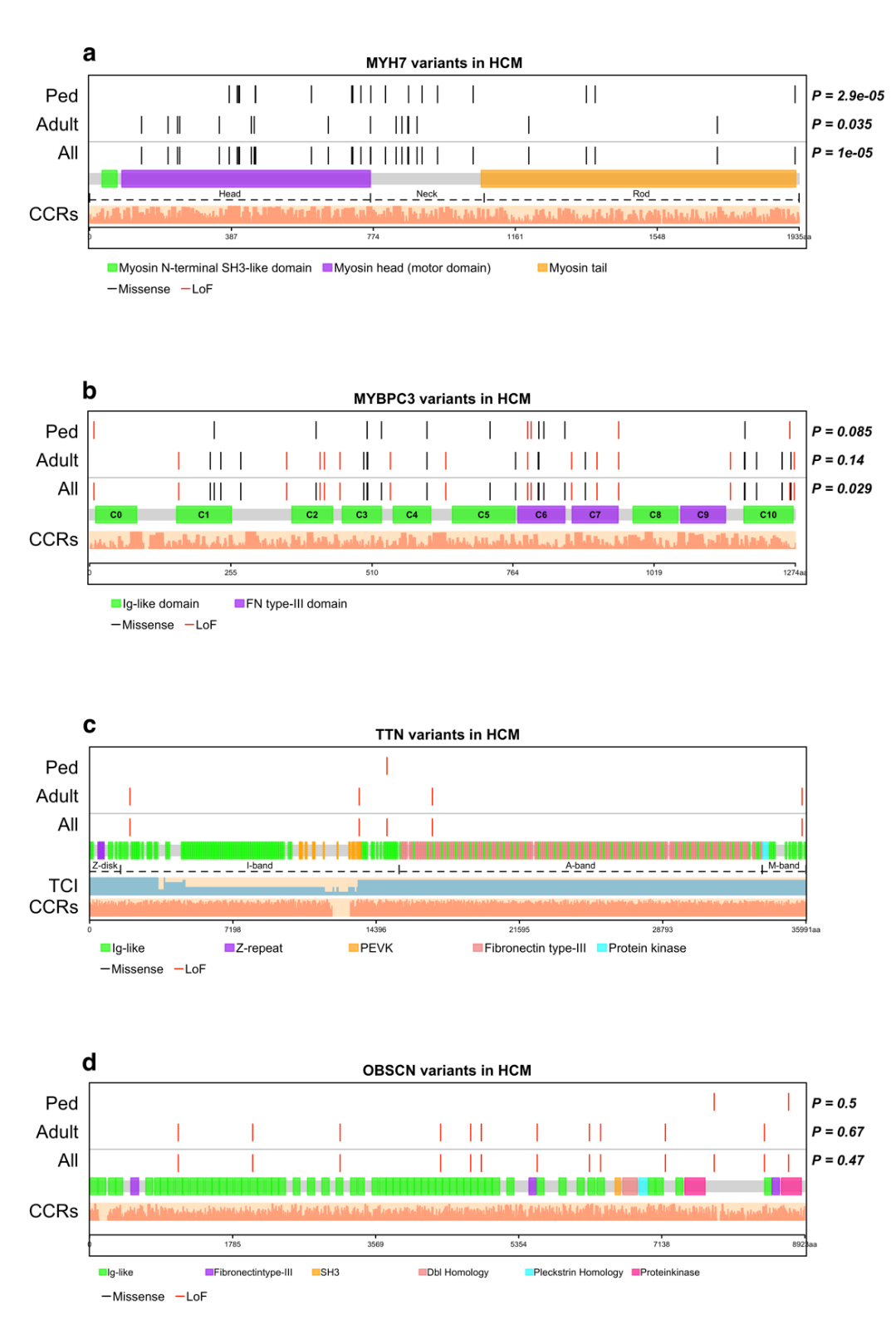


**Figure S2. Location of deleterious variants within protein domains** **in DCM stratified by age**. Each protein is linearly depicted with uniprot domain information. Non-random distribution of variants within each protein was assessed using Kolmogorov–Smirnov goodness-of-fit test. Light salmon bars show the constrained coding region scores across the entire protein. (**a**) *MYH7* showing head, neck and rod/tail regions. Deleterious variants were more frequent in the neck and tail domains in pediatric and adult patients. (**b**) *MYBPC3* showing the immunoglobulin and fibronectin type 3 domains. (**c**) *TTN* showing the four regions and all domains. The light blue bars under titin show the transcript count index. *TTN* variants clustered in the A-band particularly in adults. (**d**) *OBSCN* LoF variants were uniformly distributed across protein domains.

DCM, dilated cardiomyopathy; CCR, Constrained coding region; TCI, transcript count index; LoF, loss of function


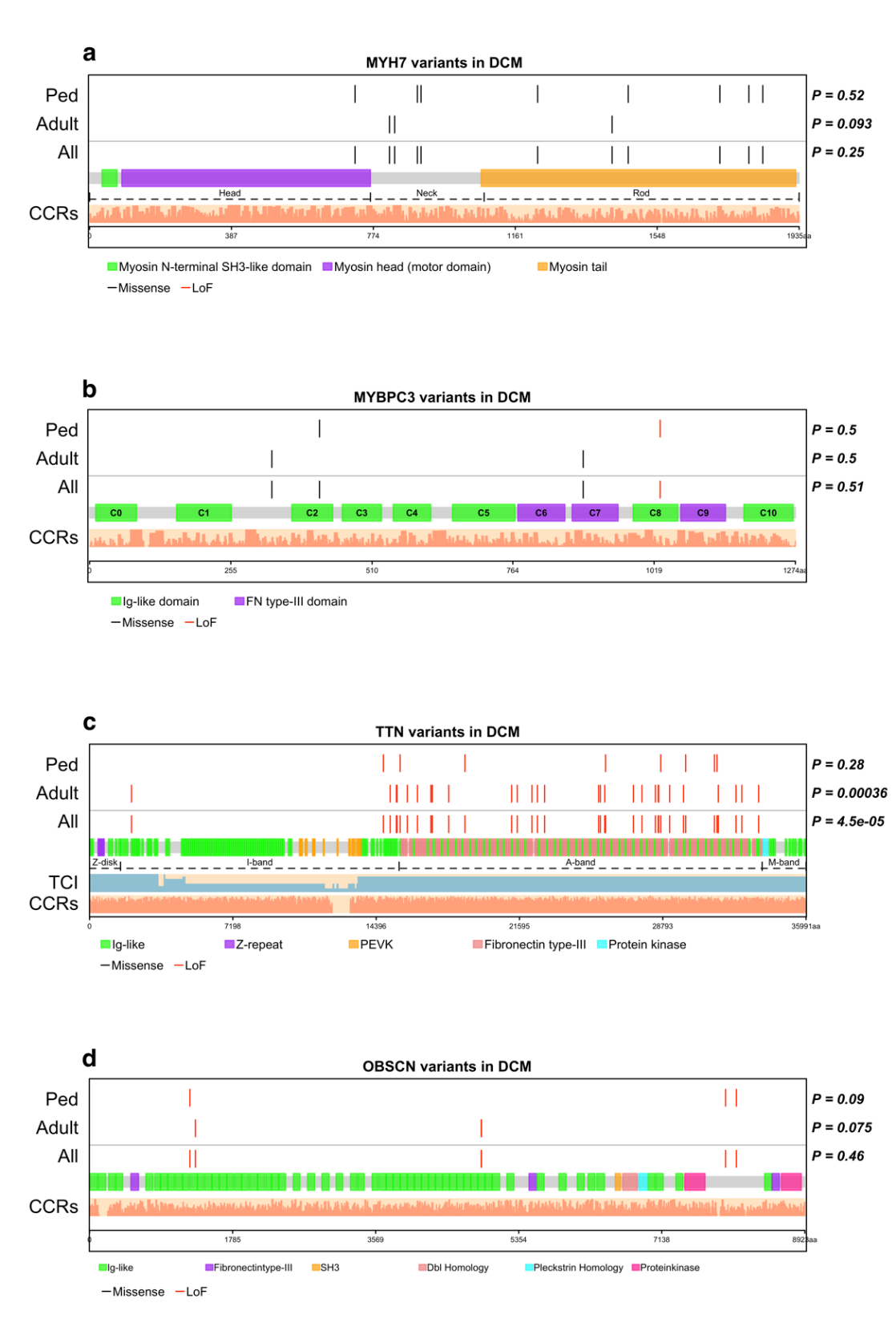


**Figure S3. Location of deleterious variants within protein domains** **in HCM stratified by sex**. Each protein is linearly depicted with uniprot domain information. Non-random distribution of variants within each protein was assessed using Kolmogorov–Smirnov goodness-of-fit test. Light salmon bars show the constrained coding region scores across the entire protein. (**a**) *MYH7* showing head, neck and rod/tail regions. Deleterious variants were non-uniformly distributed with significant clustering within head and neck domains, especially in adults. (**b**) *MYBPC3* showing the immunoglobulin and fibronectin type 3 domains. Deleterious variant distribution did not differ between pediatric and adult patients. (**c**) *TTN* showing the four regions and all domains. The light blue bars under titin show the transcript count index. (**d**) *OBSCN* LoF variants were uniformly distributed across protein domains.

HCM, hypertrophic cardiomyopathy; CCR, Constrained coding region; TCI, transcript count index; LoF, loss of function

**
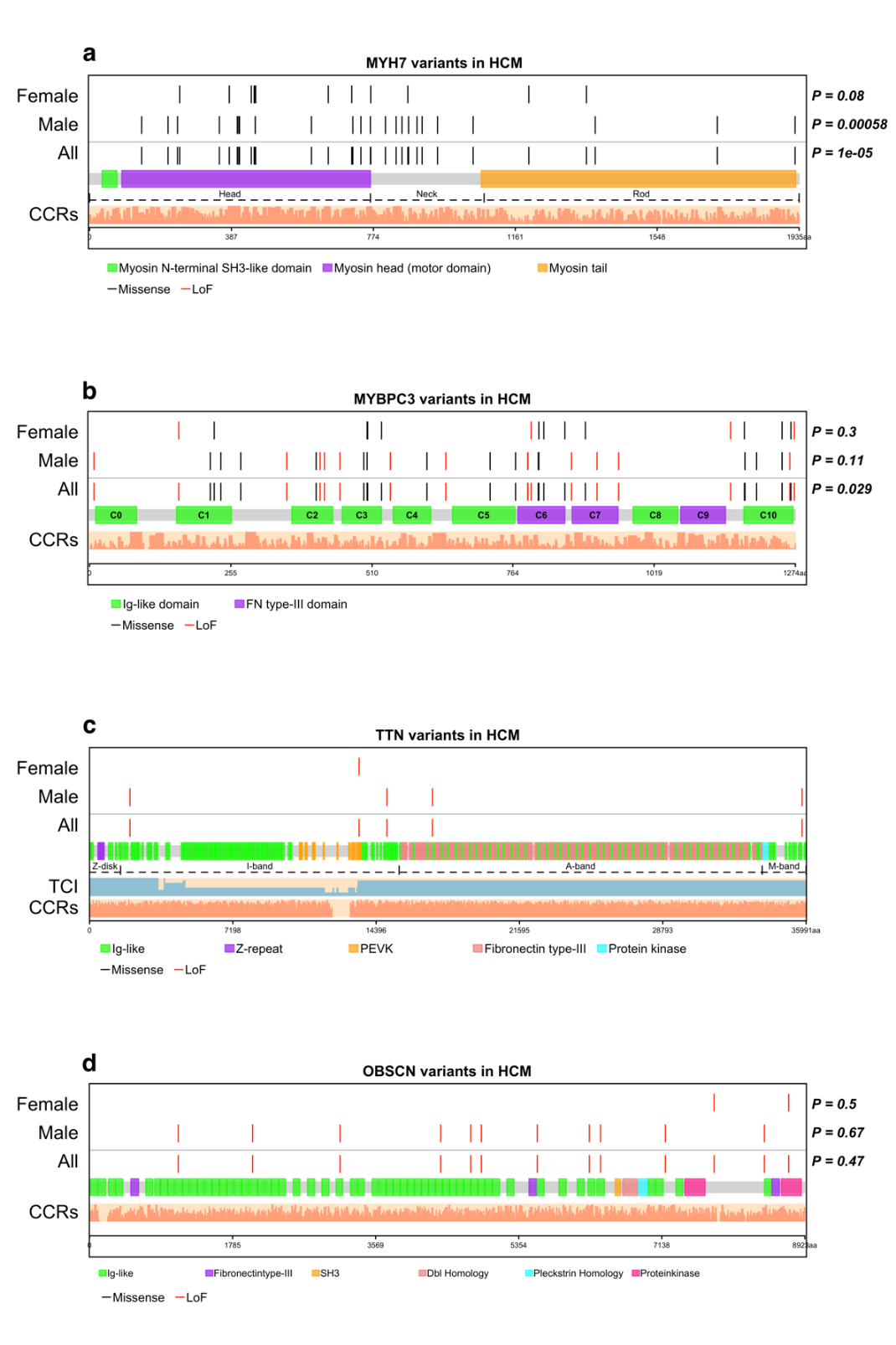
**

**Figure S4. Location of deleterious variants within protein domains** **in DCM stratified by sex**. Each protein is linearly depicted with uniprot domain information. Non-random distribution of variants within each protein was assessed using Kolmogorov–Smirnov goodness-of-fit test. Light salmon bars show the constrained coding region scores across the entire protein. (**a**) *MYH7* showing head, neck and rod/tail regions. (**b**) *MYBPC3* showing the immunoglobulin and fibronectin type 3 domains. (**c**) *TTN* showing the four regions and all domains. The light blue bars under titin show the transcript count index. *TTN* variants clustered in the A-band particularly in adults. (**d**) *OBSCN* LoF variants were uniformly distributed across protein domains.

DCM, dilated cardiomyopathy; CCR, Constrained coding region; TCI, transcript count index; LoF, loss of function

**
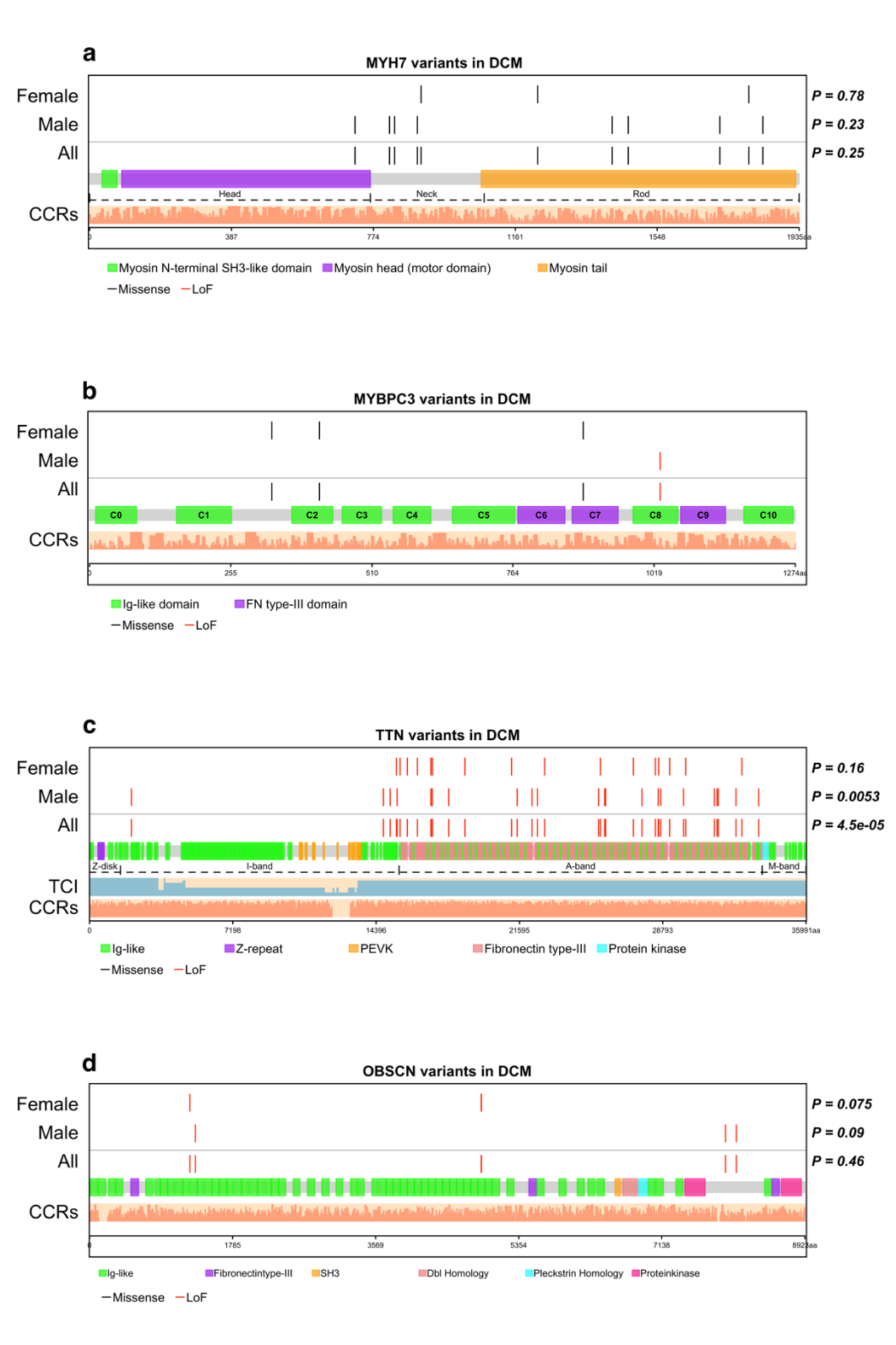
**
