# Supplementary material for: Age and Sex Differences in the Genetics of Cardiomyopathy
Source: J Cardiovasc Transl Res. 2023 Jul 21;16(6):1287–302. doi: 10.1007/s12265-023-10411-8 (PMC10721711; doi:10.1007/s12265-023-10411-8)
Supplement: Supplementary file 3 — Supplementary file3 (DOCX 118 KB) [file 12265_2023_10411_MOESM3_ESM.docx]

**SUPPLEMENTARY METHODS**

Sequencing data from the Ontario Registry are deposited in the European Genome-Phenome Archive (EGA) under accession EGAS00001004929, and are available for download upon approval by the Data Access Committee. The 100,000 Genomes Project data are available to researchers and trainees using the Genomics England Research Environment upon institutional approval through their Participation Agreement process. Additional data generated or analyzed during this study are included in the supplementary information files, and additional raw data used for figures and results are available from the corresponding author on reasonable request. Written informed consent was obtained from all biobank participants and/or their parents or legal guardians and the research protocol was approved by the Institutional Research Ethics Boards at all participating sites. The full methods are now available as supplemental data.

**Study cohort**: The study cohort comprised 1,397 unrelated primary cardiomyopathy patients recruited through (i) the multi-institutional Ontario province-wide Heart Centre Biobank Registry (n=236), and (ii) the 100,000 Genomes Project accessed through the Genomics England Clinical Interpretation Partnership (version 8) (n=1,161).^1-4^ Of these, 471 were pediatric (<18 years old at diagnosis) (236 from the Ontario biobank, 235 from Genomics England), and 926 were adults (all from Genomics England). Cardiomyopathy phenotypic subtypes were defined based on published clinical criteria.^5,6^ Patients with secondary cardiomyopathies resulting from known or suspected inborn errors of metabolism, mitochondrial disorders, chromosomal malformations, neuromuscular disorders, ischemia, and congenital heart defects were excluded. Written informed consent was obtained from all biobank participants and/or their parents or legal guardians and the research protocol was approved by the Institutional Research Ethics Boards at all participating sites.

**Whole genome sequencing and protein-coding variant interpretation**: Whole genome sequencing was performed on DNA derived from blood or saliva. The two cohorts were sequenced using a PCR-free method, on Illumina HiSeq X Ten or HiSeq 2500, with average 30X sequencing depth, and sequences were aligned to GRCh37 or GRCh38 using the Isaac Genome Alignment software as previously published.^4,7^ Whole genome sequencing quality control, data processing, and protein-coding variant identification were performed as previously described.^7,8^ Identified variants were annotated using Ensembl’s Variant Effect Predictor (VEP v92).^9^ Rare missense variants predicted damaging by SIFT and PolyPhen tools with a Combined Annotation Dependent Depletion score of at least 20 were classified as “deleterious”.^10-12^ Variants predicted to cause loss-of-function (LoF) i.e., frameshift, nonsense, and splice-site variants, were annotated by LOFTEE via VEP, and rare variants flagged as high confidence LoF by LOFTEE were classified as deleterious.^13^ Variants were defined as rare based on Minor Allele Frequency (MAF) <0.01% in aggregated data from the Genome Aggregation Database (gnomAD) v2.1.1 which includes 125,748 reference individuals with whole exome data and a subset of 15,708 individuals with whole genome sequence data.^14^ For *TTN*, only protein-truncating variants were analyzed.^15^ For the top mutated genes in the cohort (*MYH7*, *MYL3*, *OBSCN*, *TNNT2*, *TTN*, *VCL)*, a search for structural variants in protein-coding and splice regions was conducted, defined using the UCSC Table Browser.^16^ For samples obtained from the Ontario Biobank Registry, structural variants were identified using Manta, LUMPY, Wham and DELLY. ^17-20^ Variants were included only if they were called by at least 2 of the 4 tools, affected protein-coding regions of the gene, and were observed in fewer than 10 unaffected parental controls from the MSSNG cohort of 2,620 families with autism.^21^ For data from Genomics England, structural variants were identified using Manta and Canvas.^17,22^ Variants were required to have a ‘PASS’ filter and have a gnomAD structural variant v2.1 allele frequency <0.001. Inter-chromosomal translocations were excluded. Copy number losses, gains, insertions and inversions were further visually reviewed to reduce false-positive calls. Variant annotations and American College of Medical Genetics and Genomics Association for Molecular Pathology pathogenicity estimates were verified using InterVar and AutoCNV.^23-26^ Variants classified as benign or likely benign were excluded. De novo variant calling was done using Platypus.^27^ Identified de novo variants were filtered using Genomics England de novo variant filtering pipeline.

**Cardiomyopathy candidate genes**: Using information from Online Mendelian Inheritance in Man database (https://www.omim.org/), ClinGen (https://clinicalgenome.org/), published literature, and genes represented on commercially available cardiomyopathy gene panels, we manually curated a list of 78 cardiomyopathy candidate genes with autosomal dominant inheritance (**Supplementary Table S1**).^28-32^ 30 genes with definitive or moderate evidence for association with cardiomyopathy based on the above sources were classified as Tier 1, and the remainder as Tier 2. Based on gene ontology, we grouped the 78 genes into 11 functional categories and identified protein-coding variants mapping to these genes (**Supplementary Table S1**).

**Variants mapping to constrained coding regions**: Variants identified in genes with multiple transcripts were reported with respect to the canonical transcripts and were mapped to uniprot protein domains. Protein domain annotations for all canonical transcripts of cardiomyopathy genes were obtained by mining the uniprot database using a custom in house script (http://www.uniprot.org) (assessed May 28, 2020).^33^ We accessed the constrained coding region (CCR) map to obtain the CCR score for all 78 curated cardiomyopathy genes.^34^ By converting genomic coordinates to protein coordinates, we used the CCR map to obtain a measure of constraints on protein domains of cardiomyopathy genes.

**Statistical analysis**: All rare coding SNVs and indels, predicted deleterious or high risk for altering protein function and/or expression of known cardiomyopathy genes, were used in the final analysis. Variant yield and burden of multiple variants in pediatric versus adult, and male versus female cases were calculated for all cardiomyopathy patients as well as for the subset with only HCM or DCM, the two most representative cardiomyopathy subtypes. Comparisons of variant frequencies between subgroups were performed with either Chi-squared test or Fisher's exact test (limited to genes or gene categories with at least 5 variants). Ancestry was determined from the genotypes using Somalier.^35^ Additional statistical tests included a generalized linear model adjusted for gender, cardiomyopathy subtype and genetic ancestry to compare gene-level variant burden between pediatric and adult cases for genes harboring deleterious variants in the overall cohort. Benjamini–Hochberg false discovery rate correction was performed to adjust for multiple testing. Continuity correction was applied via Visualizing Categorical Data R package (v1.4-8) to estimate odds ratio and confidence interval for any zero cells in the contingency table.^36^ Variant CCR scores were compared between cases and reference control genomes available through the Genome Aggregation Database (gnomAD v2.1) (n=125,748) using Kolmogorov-Smirnov (KS) two-tailed test.^14,15,37,38^ The uniformity of the spatial distribution of deleterious variants within proteins was assessed by KS Goodness-of-Fit Test. All statistical analyses were performed using R statistical software (version 3.5.2). Using a two-proportion (2 sample, 2 sided equality) test, a sample size of 1397 provides a power of 0.78, type 1 error 5% to detect a 5% difference in variant frequency between sub-groups.

**REFERENCES**

1. Fung A, Manlhiot C, Naik S, Rosenberg H, Smythe J, Lougheed J, Mondal T, Chitayat D, McCrindle BW, Mital S. Impact of prenatal risk factors on congenital heart disease in the current era. *Journal of the American Heart Association*. 2013;2:e000064. doi: 10.1161/jaha.113.000064

2. Papaz T, Liston E, Zahavich L, Stavropoulos DJ, Jobling RK, Kim RH, Reuter M, Miron A, Oechslin E, Mondal T, et al. Return of genetic and genomic research findings: experience of a pediatric biorepository. *BMC medical genomics*. 2019;12:173. doi: 10.1186/s12920-019-0618-0

3. Papaz T, Safi M, Manickaraj AK, Ogaki C, Breaton Kyryliuk J, Burrill L, Dodge C, Chant-Gambacort C, Walter LL, Rosenberg H, et al. Factors influencing participation in a population-based biorepository for childhood heart disease. *Pediatrics*. 2012;130:e1198-1205. doi: 10.1542/peds.2012-0687

4. Smedley D, Smith KR, Martin A, Thomas EA, McDonagh EM, Cipriani V, Ellingford JM, Arno G, Tucci A, Vandrovcova J, et al. 100,000 Genomes Pilot on Rare-Disease Diagnosis in Health Care - Preliminary Report. *The New England journal of medicine*. 2021;385:1868-1880. doi: 10.1056/NEJMoa2035790

5. Elliott P, Andersson B, Arbustini E, Bilinska Z, Cecchi F, Charron P, Dubourg O, Kühl U, Maisch B, McKenna WJ, et al. Classification of the cardiomyopathies: a position statement from the European Society Of Cardiology Working Group on Myocardial and Pericardial Diseases. *European heart journal*. 2008;29:270-276. doi: 10.1093/eurheartj/ehm342

6. Maron BJ, Towbin JA, Thiene G, Antzelevitch C, Corrado D, Arnett D, Moss AJ, Seidman CE, Young JB. Contemporary definitions and classification of the cardiomyopathies: an American Heart Association Scientific Statement from the Council on Clinical Cardiology, Heart Failure and Transplantation Committee; Quality of Care and Outcomes Research and Functional Genomics and Translational Biology Interdisciplinary Working Groups; and Council on Epidemiology and Prevention. *Circulation*. 2006;113:1807-1816. doi: 10.1161/circulationaha.106.174287

7. Lesurf R, Said A, Akinrinade O, Breckpot J, Delfosse K, Liu T, Yao R, Persad G, McKenna F, Noche RR, et al. Whole genome sequencing delineates regulatory, copy number, and cryptic splice variants in early onset cardiomyopathy. *NPJ genomic medicine*. 2022;7:18. doi: 10.1038/s41525-022-00288-y

8. Yao RA, Akinrinade O, Chaix M, Mital S. Quality of whole genome sequencing from blood versus saliva derived DNA in cardiac patients. *BMC medical genomics*. 2020;13:11. doi: 10.1186/s12920-020-0664-7

9. McLaren W, Gil L, Hunt SE, Riat HS, Ritchie GR, Thormann A, Flicek P, Cunningham F. The Ensembl Variant Effect Predictor. *Genome biology*. 2016;17:122. doi: 10.1186/s13059-016-0974-4

10. Adzhubei I, Jordan DM, Sunyaev SR. Predicting functional effect of human missense mutations using PolyPhen-2. *Current protocols in human genetics*. 2013;Chapter 7:Unit7.20. doi: 10.1002/0471142905.hg0720s76

11. Kircher M, Witten DM, Jain P, O'Roak BJ, Cooper GM, Shendure J. A general framework for estimating the relative pathogenicity of human genetic variants. *Nature genetics*. 2014;46:310-315. doi: 10.1038/ng.2892

12. Ng PC, Henikoff S. SIFT: Predicting amino acid changes that affect protein function. *Nucleic acids research*. 2003;31:3812-3814. doi: 10.1093/nar/gkg509

13. Petrovski S, Wang Q, Heinzen EL, Allen AS, Goldstein DB. Genic intolerance to functional variation and the interpretation of personal genomes. *PLoS genetics*. 2013;9:e1003709. doi: 10.1371/journal.pgen.1003709

14. Karczewski KJ, Francioli LC, Tiao G, Cummings BB, Alföldi J, Wang Q, Collins RL, Laricchia KM, Ganna A, Birnbaum DP, et al. The mutational constraint spectrum quantified from variation in 141,456 humans. *Nature*. 2020;581:434-443. doi: 10.1038/s41586-020-2308-7

15. Herman DS, Lam L, Taylor MR, Wang L, Teekakirikul P, Christodoulou D, Conner L, DePalma SR, McDonough B, Sparks E, et al. Truncations of titin causing dilated cardiomyopathy. *The New England journal of medicine*. 2012;366:619-628. doi: 10.1056/NEJMoa1110186

16. Karolchik D, Hinrichs AS, Furey TS, Roskin KM, Sugnet CW, Haussler D, Kent WJ. The UCSC Table Browser data retrieval tool. *Nucleic acids research*. 2004;32:D493-496. doi: 10.1093/nar/gkh103

17. Chen X, Schulz-Trieglaff O, Shaw R, Barnes B, Schlesinger F, Källberg M, Cox AJ, Kruglyak S, Saunders CT. Manta: rapid detection of structural variants and indels for germline and cancer sequencing applications. *Bioinformatics (Oxford, England)*. 2016;32:1220-1222. doi: 10.1093/bioinformatics/btv710

18. Layer RM, Chiang C, Quinlan AR, Hall IM. LUMPY: a probabilistic framework for structural variant discovery. *Genome biology*. 2014;15:R84. doi: 10.1186/gb-2014-15-6-r84

19. Kronenberg ZN, Osborne EJ, Cone KR, Kennedy BJ, Domyan ET, Shapiro MD, Elde NC, Yandell M. Wham: Identifying Structural Variants of Biological Consequence. *PLoS computational biology*. 2015;11:e1004572. doi: 10.1371/journal.pcbi.1004572

20. Rausch T, Zichner T, Schlattl A, Stütz AM, Benes V, Korbel JO. DELLY: structural variant discovery by integrated paired-end and split-read analysis. *Bioinformatics (Oxford, England)*. 2012;28:i333-i339. doi: 10.1093/bioinformatics/bts378

21. RK CY, Merico D, Bookman M, J LH, Thiruvahindrapuram B, Patel RV, Whitney J, Deflaux N, Bingham J, Wang Z, et al. Whole genome sequencing resource identifies 18 new candidate genes for autism spectrum disorder. *Nature neuroscience*. 2017;20:602-611. doi: 10.1038/nn.4524

22. Roller E, Ivakhno S, Lee S, Royce T, Tanner S. Canvas: versatile and scalable detection of copy number variants. *Bioinformatics (Oxford, England)*. 2016;32:2375-2377. doi: 10.1093/bioinformatics/btw163

23. Richards S, Aziz N, Bale S, Bick D, Das S, Gastier-Foster J, Grody WW, Hegde M, Lyon E, Spector E, et al. Standards and guidelines for the interpretation of sequence variants: a joint consensus recommendation of the American College of Medical Genetics and Genomics and the Association for Molecular Pathology. *Genetics in medicine : official journal of the American College of Medical Genetics*. 2015;17:405-424. doi: 10.1038/gim.2015.30

24. Riggs ER, Andersen EF, Cherry AM, Kantarci S, Kearney H, Patel A, Raca G, Ritter DI, South ST, Thorland EC, et al. Technical standards for the interpretation and reporting of constitutional copy-number variants: a joint consensus recommendation of the American College of Medical Genetics and Genomics (ACMG) and the Clinical Genome Resource (ClinGen). *Genetics in medicine : official journal of the American College of Medical Genetics*. 2020;22:245-257. doi: 10.1038/s41436-019-0686-8

25. Li Q, Wang K. InterVar: Clinical Interpretation of Genetic Variants by the 2015 ACMG-AMP Guidelines. *American journal of human genetics*. 2017;100:267-280. doi: 10.1016/j.ajhg.2017.01.004

26. Fan C, Wang Z, Sun Y, Sun J, Liu X, Kang L, Xu Y, Yang M, Dai W, Song L, et al. AutoCNV: a semiautomatic CNV interpretation system based on the 2019 ACMG/ClinGen Technical Standards for CNVs. *BMC genomics*. 2021;22:721. doi: 10.1186/s12864-021-08011-4

27. Rimmer A, Phan H, Mathieson I, Iqbal Z, Twigg SRF, Wilkie AOM, McVean G, Lunter G. Integrating mapping-, assembly- and haplotype-based approaches for calling variants in clinical sequencing applications. *Nature genetics*. 2014;46:912-918. doi: 10.1038/ng.3036

28. Ingles J, Goldstein J, Thaxton C, Caleshu C, Corty EW, Crowley SB, Dougherty K, Harrison SM, McGlaughon J, Milko LV, et al. Evaluating the Clinical Validity of Hypertrophic Cardiomyopathy Genes. *Circulation Genomic and precision medicine*. 2019;12:e002460. doi: 10.1161/circgen.119.002460

29. Jordan E, Peterson L, Ai T, Asatryan B, Bronicki L, Brown E, Celeghin R, Edwards M, Fan J, Ingles J, et al. Evidence-Based Assessment of Genes in Dilated Cardiomyopathy. *Circulation*. 2021;144:7-19. doi: 10.1161/circulationaha.120.053033

30. Mazzarotto F, Tayal U, Buchan RJ, Midwinter W, Wilk A, Whiffin N, Govind R, Mazaika E, de Marvao A, Dawes TJW, et al. Reevaluating the Genetic Contribution of Monogenic Dilated Cardiomyopathy. *Circulation*. 2020;141:387-398. doi: 10.1161/circulationaha.119.037661

31. Thomson KL, Ormondroyd E, Harper AR, Dent T, McGuire K, Baksi J, Blair E, Brennan P, Buchan R, Bueser T, et al. Analysis of 51 proposed hypertrophic cardiomyopathy genes from genome sequencing data in sarcomere negative cases has negligible diagnostic yield. *Genetics in medicine : official journal of the American College of Medical Genetics*. 2019;21:1576-1584. doi: 10.1038/s41436-018-0375-z

32. Walsh R, Buchan R, Wilk A, John S, Felkin LE, Thomson KL, Chiaw TH, Loong CCW, Pua CJ, Raphael C, et al. Defining the genetic architecture of hypertrophic cardiomyopathy: re-evaluating the role of non-sarcomeric genes. *European heart journal*. 2017;38:3461-3468. doi: 10.1093/eurheartj/ehw603

33. Consortium U. UniProt: a worldwide hub of protein knowledge. *Nucleic acids research*. 2019;47:D506-d515. doi: 10.1093/nar/gky1049

34. Havrilla JM, Pedersen BS, Layer RM, Quinlan AR. A map of constrained coding regions in the human genome. *Nature genetics*. 2019;51:88-95. doi: 10.1038/s41588-018-0294-6

35. Pedersen BS, Bhetariya PJ, Brown J, Kravitz SN, Marth G, Jensen RL, Bronner MP, Underhill HR, Quinlan AR. Somalier: rapid relatedness estimation for cancer and germline studies using efficient genome sketches. *Genome medicine*. 2020;12:62. doi: 10.1186/s13073-020-00761-2

36. Kim J, Zhang Y, Day J, Zhou H. MGLM: An R Package for Multivariate Categorical Data Analysis. *The R journal*. 2018;10:73-90. doi: 10.32614/rj-2018-015

37. Akinrinade O, Alastalo TP, Koskenvuo JW. Relevance of truncating titin mutations in dilated cardiomyopathy. *Clinical genetics*. 2016;90:49-54. doi: 10.1111/cge.12741

38. Roberts AM, Ware JS, Herman DS, Schafer S, Baksi J, Bick AG, Buchan RJ, Walsh R, John S, Wilkinson S, et al. Integrated allelic, transcriptional, and phenomic dissection of the cardiac effects of titin truncations in health and disease. *Science translational medicine*. 2015;7:270ra276. doi: 10.1126/scitranslmed.3010134
